# Three generations of mTOR kinase inhibitors in the activation of the apoptosis process in melanoma cells

**DOI:** 10.1007/s12079-023-00748-9

**Published:** 2023-04-25

**Authors:** Dorota Ciołczyk-Wierzbicka, Agnieszka Krawczyk, Marta Zarzycka, Grzegorz Zemanek, Karol Wierzbicki

**Affiliations:** 1 Chair of Medical Biochemistry, Jagiellonian University Medical College, Ul. Kopernika 7, 31-034 Kraków, Poland; 2 Department of Cardiovascular Surgery and Transplantology, Institute of Cardiology, Jagiellonian University, John Paul II Hospital, Ul. Prądnicka 80, 31-202 Kraków, Poland

**Keywords:** mTOR inhibitors, Apoptosis, Caspase-3 activity, Proliferation, Melanoma, Immunosuppressive treatment

## Abstract

Many signaling pathways are involved in the mammalian target of rapamycin (mTOR), and this serine/threonine kinase regulates the most important cellular processes such as cell proliferation, autophagy, and apoptosis. The subject of this research was the effect of protein kinase inhibitors involved in the AKT, MEK, and mTOR kinase signaling pathways on the expression of pro-survival proteins, activity of caspase-3, proliferation, and induction of apoptosis in melanoma cells. The following inhibitors were used: protein kinase inhibitors such as AKT—MK-2206, MEK—AS-703026, mTOR—everolimus and Torkinib, as well as dual PI3K and mTOR inhibitor—BEZ-235 and Omipalisib, and mTOR1/2—OSI-027 inhibitor in single-mode and their combinations with MEK1/2 kinase inhibitor AS-703026. The obtained results confirm the synergistic effect of nanomolar concentrations of mTOR inhibitors, especially the dual PI3K and mTOR inhibitors (Omipalisib, BEZ-235) in combination with the MAP kinase inhibitor (AS-703026) in the activation of caspase 3, induction of apoptosis, and inhibition of proliferation in melanoma cell lines. Our previous and current studies confirm the importance of the mTOR signal transduction pathway in the neoplastic transformation process. Melanoma is a case of a very heterogeneous neoplasm, which causes great difficulties in treating this neoplasm in an advanced stage, and the standard approach to this topic does not bring the expected results. There is a need for research on the search for new therapeutic strategies aimed at particular groups of patients.

**Graphical abstract:**

Effect of three generations of mTOR kinase inhibitors on caspase-3 activity, apoptosis and proliferation in melanoma cell lines.
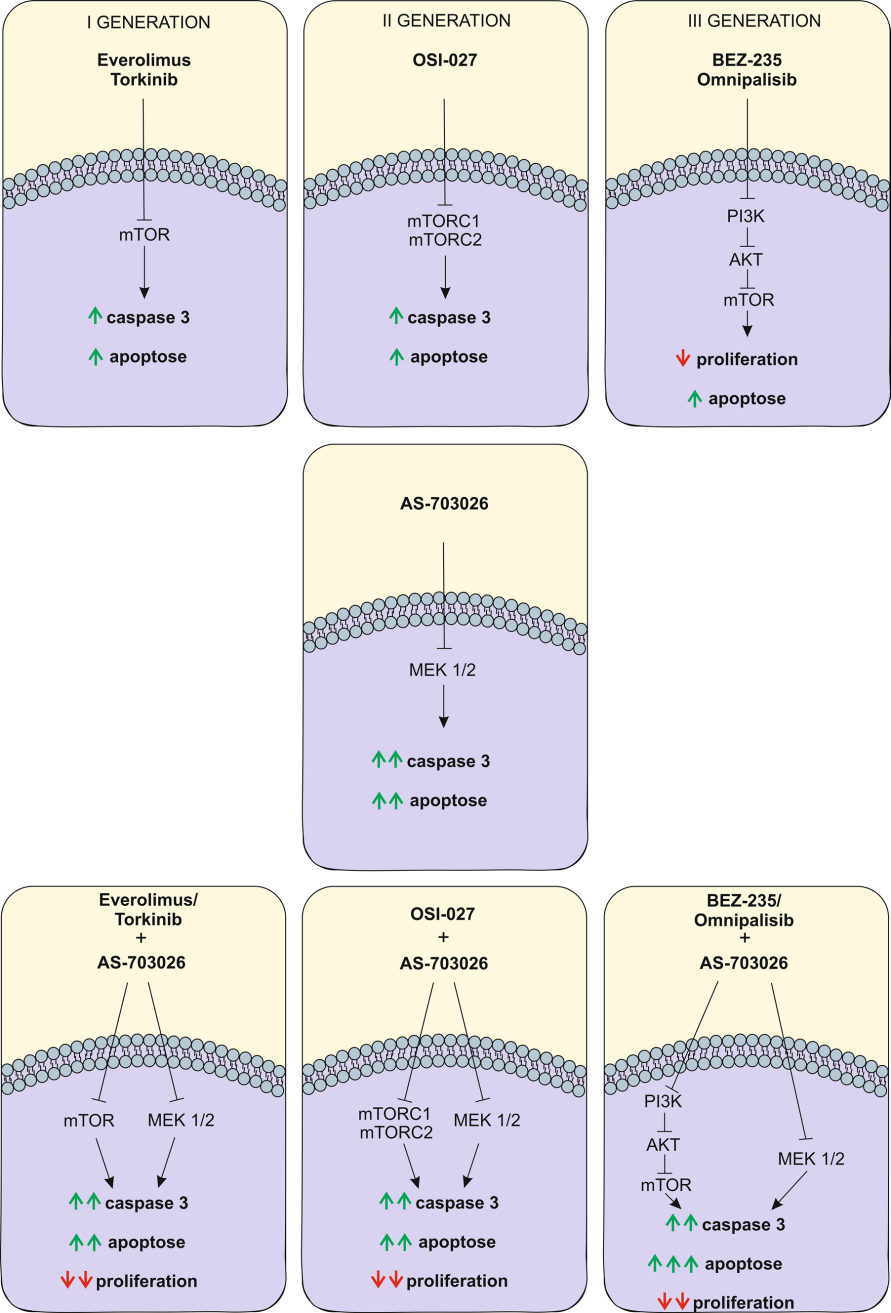

**Supplementary Information:**

The online version contains supplementary material available at 10.1007/s12079-023-00748-9.

## Introduction

mTOR kinase exists as two functionally separate protein complexes: mTORC1 (mammalian target of the rapamycin complex 1) and mTORC2 (mammalian target of the rapamycin complex 2), each of which has a different subcellular localization that also determines its distinct functions and independent regulations (Zou et al. 2020). mTORC1 is associated with endosomal and lysosomal membranes, where it interacts through phosphorylation with its effectors, the two best characterized substrates: S6 ribosomal kinase (S6K1, ribosomal protein S6 kinase 1) and eukaryotic binding protein translation initiation factor 4E (4E-BP1, Eukaryotic translation initiation factor 4E-binding protein 1). The mTORC1 complex initiates the translation of proteins essential for cell cycle progression and the synthesis of nucleotides and lipids. It also participates in lysosomal biogenesis and suppression of the autophagy process (Zou et al. 2020). mTORC2, on the other hand, is affiliated with the plasma membrane. Its molecular mechanism is yet to be clarified. The primary functions of the mTORC2 complex appear to be the organization of the cytoskeleton, cell migration, inhibition of apoptosis, and affecting metabolism (Zou et al. 2020).

Furthermore, the mTORC3 complex, which is insensitive to rapamycin and plays a role in the proliferation process (Hage and Dormond 2021), has been recently mentioned.

Regulation of the mTOR-related signaling pathway is much more complicated than it initially appeared. It can be influenced by its positive and negative regulators, such as phosphoinositide 3-kinase (PI3K)/Akt, mitogen-activated protein kinase (MAPK), vascular endothelial growth factor (VEGF), nuclear factor-κB (NF-κB), and p53 (He et al. 2021).

Dysregulation of the mTOR signal is illustrated in many human diseases, especially in various cancers. Data in the literature show that overexpression of mTOR kinase occurs in approximately 70% of cancer cases (Tian et al. 2019).

Interest in mTOR inhibitors dates back to the 1970s, when rapamycin was isolated from the fungus Streptomyces hygroscopius. Initially, the mTOR inhibitors rapamycin and its rapalogs found their application in immunosuppressive therapy after organ transplantation. Later, because the mTOR signaling pathway is active in most human cancers, the first- and second-generation mTOR inhibitors for cancer treatment were developed (Hage and Dormond 2021).

Several types of mTOR inhibitors, the first- and second-generation, have been examined in various cancer models, including breast, lung, gastric, colorectal, prostate, head and neck, lymphoma, urinary bladder, renal, and melanoma (Zou et al. 2020; Li et al. 2018). The first-generation mTOR kinase inhibitors, such as temsirolimus, everolimus, have been approved by the U.S. Food and Drug Administration for use in kidney or breast cancer.

The promising results of our previous research (Ciołczyk-Wierzbicka and Laidler 2018; Ciołczyk-Wierzbicka et al. 2018, 2019, 2020) on inhibition of invasion and activation of the apoptosis process with the use of nanomolar concentrations of everolimus, the first-generation mTOR inhibitor, encouraged us to investigate the effect of three generations of mTOR kinase inhibitors on activation of caspase 3, the apoptosis process, and proliferation in melanoma cells.

Melanoma represents a very heterogeneous neoplasm, which makes treating its advanced stage difficult, and the standard approach to this topic not as effective as expected.

The search for new treatment regimens tailored for individual patient groups is still an open path, and for this purpose, among others, basic research on models based on cell lines is being carried out.

## Materials and methods

### Cell culture

In the presented study, three melanoma cell lines were used: primary (VGP)—WM3211 and metastatic: Mel 1359 and MEWO. WM3211 human primary melanoma cell line (VGP) with metastasis competence, wild type for BRAF, PTEN, N-RAS, and CDK4, and with mutation at position 576 in the c-KIT gene.

Mel-1359 human malignant melanoma cell line. This cell line features the specific V600E mutation in the BRAF gene and the wild type of PTEN, N-RAS. MEWO, human malignant melanoma cell line derived from the lymph node. This cell line is wild type for BRAF, PTEN, and N-RAS.

The cells were cultured in RPMI-1640 medium supplemented with 10% fetal bovine serum and antibiotics: penicillin and streptomycin. The cells were incubated at 37 °C in a humidified atmosphere of 5% CO2 in air. The cells were treated with inhibitors of 1/AKT—MK-2206 (Selleck) at 2 μM concentration, 2/MEK1/2—AS-703026 (Selleck) at 10 μM concentration, 3/mTOR—everolimus (Selleck) at 20 nM concentration, 4/dual PI3K and mTOR inhibitor—BEZ-235 (NVP-BEZ235), Selleck) at 20 nM, 5/dual PI3K and mTOR inhibitor—Omipalisib (GSK2126458), Selleck) at 20 nM concentration, 6/mTOR1/2—OSI-027 (Selleck) at 20 nM concentration and 7/mTOR—Torkinib (PP242), Selleck) at 20 nM concentration. The incubation time of the melanoma cells with inhibitors was 24 and 48 h. The cells were obtained from the ESTDAB Melanoma Cell Bank (Tübingen, Germany).

### Caspase-3 activation assay

Activation of caspase-3 in response to the applied inhibitors was estimated using the fluorogenic substrate DEVD-AFC (Biovision) as previously described (Ciołczyk-Wierzbicka et al. 2019).

### DNA fragmentation ELISA assay

Apoptosis induction was verified by assessing the intracellular level of DNA fragments associated with cytoplasmic histone-associated-DNA-fragments (mono- and oligonucleosomes) that appear after caspase-dependent endonucleases activation using enzyme immunoassay as previously described (Ciołczyk-Wierzbicka et al. 2019).

### Cell proliferation

Cell proliferation was assessed with the crystal violet test as previously described (Ciołczyk-Wierzbicka et al. 2019).

### Cytotoxicity assay

Cytotoxicity of selected kinase inhibitors: AKT—MK-2206 (2 μM), MEK1/2—AS-703026 (10 μM), mTOR—everolimus (20 nM), dual PI3K and mTOR inhibitor—BEZ-235 (20 nM), dual PI3K and mTOR inhibitor—Omipalisib (20 nM), mTOR1/2—OSI-027 (20 nM), mTOR—Torkinib (20 nM) was determined using Cytotoxicity Detection Kit LDH, Roche, Germany.

### Western blot analysis

Samples for SDS-PAGE electrophoresis were prepared as previously described (Ciolczyk-Wierzbicka et al. 2012). Antibodies against: Phospho-Bcl-2 (Thr56) #2875, Bcl-2 (D55G8) 4223#, Bcl-xl (54H6) 2764#, Mcl-1 (D35A5) 5453#, phospho-mTOR (Ser2448) (D9C2) #5536, phospho-mTOR (Ser2481) (cell signaling technology) and β-actin (A2228, SIGMA) were used to detect the indicated proteins. Bands were visualized using horseradish peroxidase-coupled secondary antimouse or antirabbit antibody (Cell Signaling Technology). The immunoreactivity of the protein was detected by chemiluminescence and images were captured with a ChemiDoc MP Imaging System (Bio-Rad Labs). To obtain quantitative results, immunoblots were scanned using SynGene Gene Tools version 4.03.0 (Synoptics Ltd Beacon House, Nuffield Road Cambridge, CB4 1TF, UK). Densitometry was performed to normalized β-actin protein level. Representative membranes of at least three independent experiments are presented.

### DAPI staining assay

4′,6-diamidino-2-phenylindole (DAPI, Roche) staining was carried out according to the method described by manual protocol 4′,6-Diamidine-2′-phenylindole dihydrochloride Cat. No. 10 236 276 001 (Roche).

### Densitometry analysis

Band densitometry analyses in western blot analysis were performed on raw volume (sum of intensities of the bound volume calculated from the area of the peak) using SynGene Gene Tools version 4.03.0 (Synoptics Ltd Beacon House, Nuffield Road Cam-bridge, CB4 1TF, UK).

Densitometry was performed to the normalized β-actin protein level. Presented are representative membranes of at least three independent experiments with similar results.

### Statistics

The data on caspase-3 activity, proliferation, and apoptosis were calculated from the mean values of repeated experiments. Statistical analyses were performed using one-way ANOVA with a post hoc Dunett test (Statistica 12.0 StatSoft). The statistical significance is presented in the relevant figures.

## Results

### Effect of mTOR inhibitors on the expression of pro-survival proteins

**Fig. 1:**
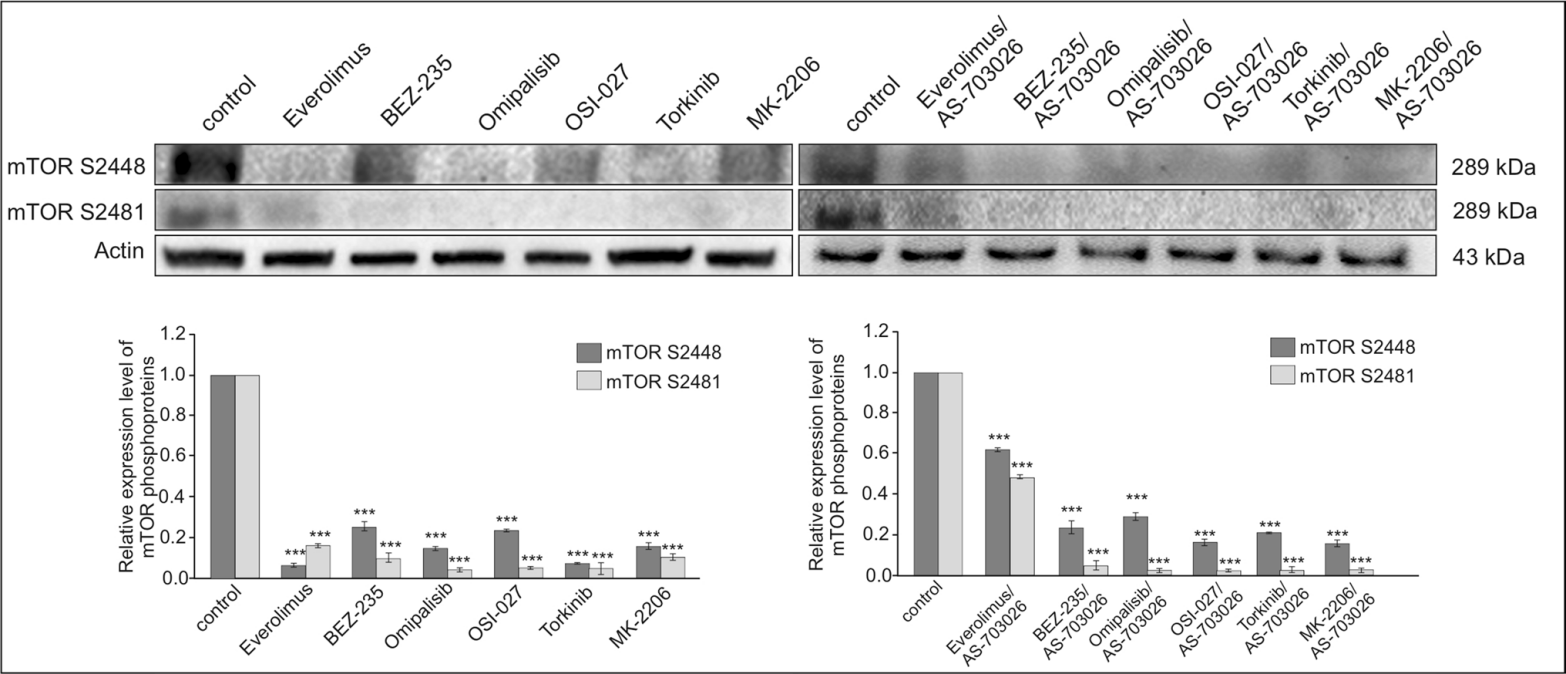
Effect of mTOR inhibitors on the expression of phospho-mTOR (Ser2441) and Phospho-mTOR (Ser2448). Actin was used as a loading control. The densitometric analysis of phospho-mTOR (Ser2441) and Phospho-mTOR (Ser2448) was normalized against its corresponding β-actin data point. The data obtained from three separate analyses are expressed as mean ± SD. Statistical analyses were performed using one-way ANOVA with a post hoc Dunett test (Statistica 12.0 StatSoft); significant differences from control values are indicated as (*) *p* < 0.05, (**) *p* < 0.01, (***) *p* < 0.001

We investigated the effect of three generations of mTOR kinase inhibitors alone and in combination with the MEK1/2 kinase inhibitor AS-703026 on the expression of pro-survival proteins: p-Bcl-2 (T56), Bcl-2, Bcl-xL, and Mcl-1 in MEWO melanoma cell line. All the used mTOR kinase inhibitors, both of the first generation—everolimus and Torkinib, as well as the mTOR 1/2 -OSI-027 inhibitor, and dual PI3K and mTOR inhibitor, BEZ-235, Omipalisib, inhibited the expression of the phosphorylated form of phospho-mTOR (Ser2481) almost completely and significantly inhibited forms of phospho-mTOR (Ser2448) (Fig. 1). The AKT kinase inhibitor—MK-2206 showed similar results. The effect was even more pronounced for the combination of mTOR inhibitors with the MEK1/2 inhibitor—AS-703026 (Fig. 1).

**Fig. 2:**
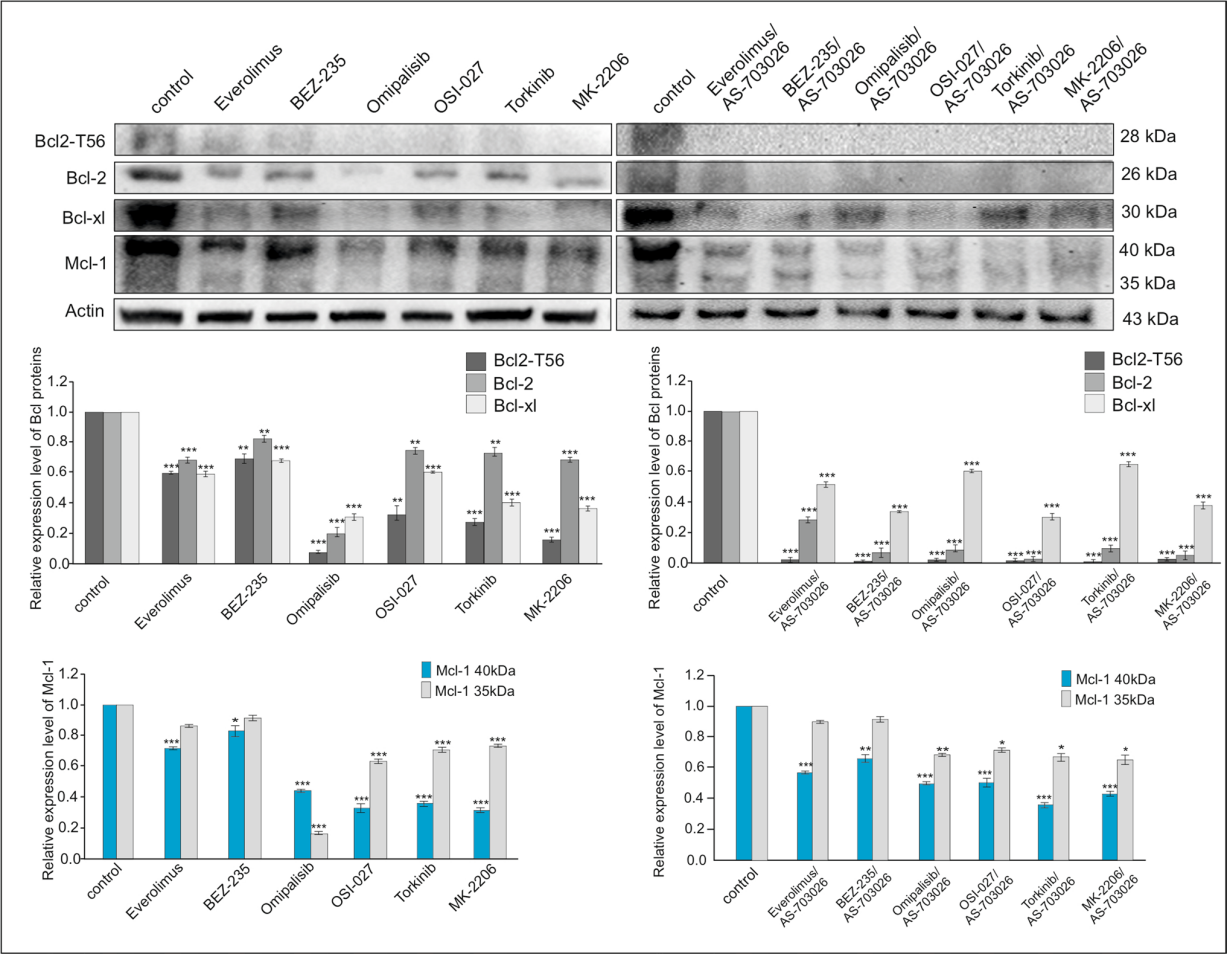
Effect of the combination of mTOR inhibitors with the MEK1/2 inhibitor AS-703026 on the expression of pro-survival proteins in MEWO melanoma cell lines. Expression of pro-survival proteins. Actin was used as a loading control. The densitometric analysis of pro-survival protein was normalized against its corresponding β-actin data point. The data obtained from three separate analyses are expressed as mean ± SD. Statistical analyses were performed using one-way ANOVA with a post hoc Dunett test (Statistica 12.0 StatSoft); significant differences from control values are indicated as (*) *p* < 0.05, (**) *p* < 0.01, (***) *p* < 0.001

The used mTOR kinase inhibitors decreased the expression of pro-survival proteins significantly; the addition of the combination with the MEK1/2—AS-703026 inhibitor resulted in an additional synergistic effect in inhibiting the expression of Bcl-2 family proteins (Fig. 2).

The expression of the p-Bcl-2 (T56) protein decreased by about 80% after the use of Omipalisib inhibitors; a slightly less pronounced effect was observed for the everolimus inhibitor and the dual inhibitor of the mTOR and AKT pathway, BEZ-235 (Fig. 2). Bcl-2 protein levels also decreased significantly (Fig. 2). The use of a combination of mTOR inhibitors with the MEK1/2 inhibitor AS-703026 almost stopped the expression of these proteins (Fig. 2).

The Bcl-xL protein level was also reduced to approximately 40–60%, in which case the AS-703026 inhibitor showed no clear enhancing effect (Fig. 2). On the other hand, for the Mcl-1 protein, a weaker decrease in expression was obtained after the use of mTOR kinase inhibitors at the level of 40%; the use of combination with an AS-703026 inhibitor did not result in an enhancement (Fig. 2).

### Effect of mTOR and PI3K kinase inhibitors on caspase-3 activity and proliferation

We investigated the effect of protein kinase inhibitors involved in the AKT, MEK, and mTOR kinase signaling pathways on caspase-3 activation and proliferation in WM3211, Mel-1359, and MEWO melanoma cell lines. We used protein kinase inhibitors such as AKT—MK-2206, MEK1/2—AS-703026, mTOR—everolimus and Torkinib, mTOR1/2—OSI-027 inhibitor, as well as dual PI3K and mTOR inhibitor—BEZ-235, and Omipalisib in single mode, and their combinations with the MEK1/2 kinase inhibitor AS-703026.

**Fig. 3:**
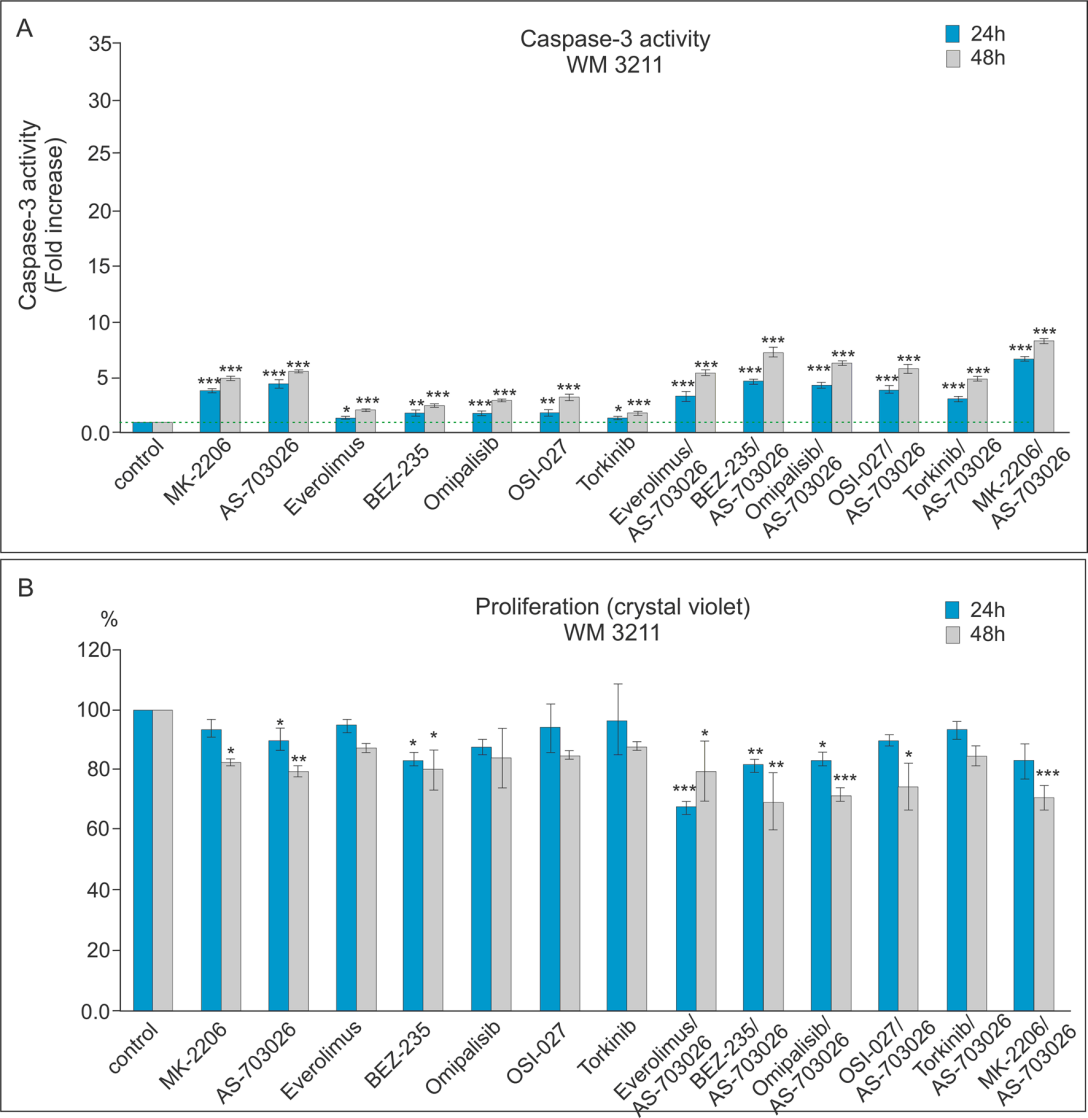
Effect of mTOR kinase inhibitors on caspase-3 activity (**A**) and cell proliferation in WM 3211 melanoma cell lines (**B**). Caspase-3 activity (**A**) and cell proliferation—crystal violet assay (**B**) were calculated from the mean values of three independent experiments. Each value was expressed as a ratio of caspase-3 activity or cell proliferation level to the control level; the control value was set to 1 for caspase-3 activity and 100% for cell proliferation. The data are presented as mean ± standard deviation; Statistical analyses were performed using one-way ANOVA with a post hoc Dunett test (Statistica 12.0 StatSoft); significant differences from control values are indicated as (*) *p* < 0.05, (**) *p* < 0.01, (***) *p* < 0.001

**Fig. 4:**
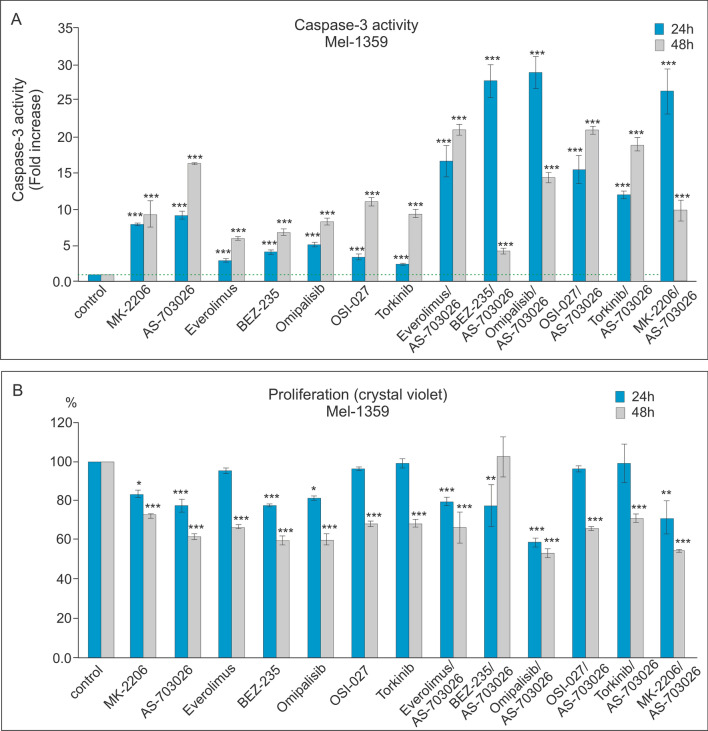
Effect of mTOR kinase inhibitors on caspase-3 activity (**A**) and cell proliferation in Mel-1359 melanoma cell lines (B). Caspase-3 activity (**A**) and cell proliferation—crystal violet assay (**B**) were calculated from the mean values of three independent experiments. Each value was expressed as the ratio of caspase-3 activity or cell proliferation level to the control level; the control value was set at 1 for caspase-3 activity and 100% for cell proliferation. The data are presented as mean ± standard deviation; Statistical analyses were performed using one-way ANOVA with a post hoc Dunett test (Statistica 12.0 StatSoft); significant differences from control values are indicated as (*) *p* < 0.05, (**) *p* < 0.01, (***) *p* < 0.001

**Fig. 5:**
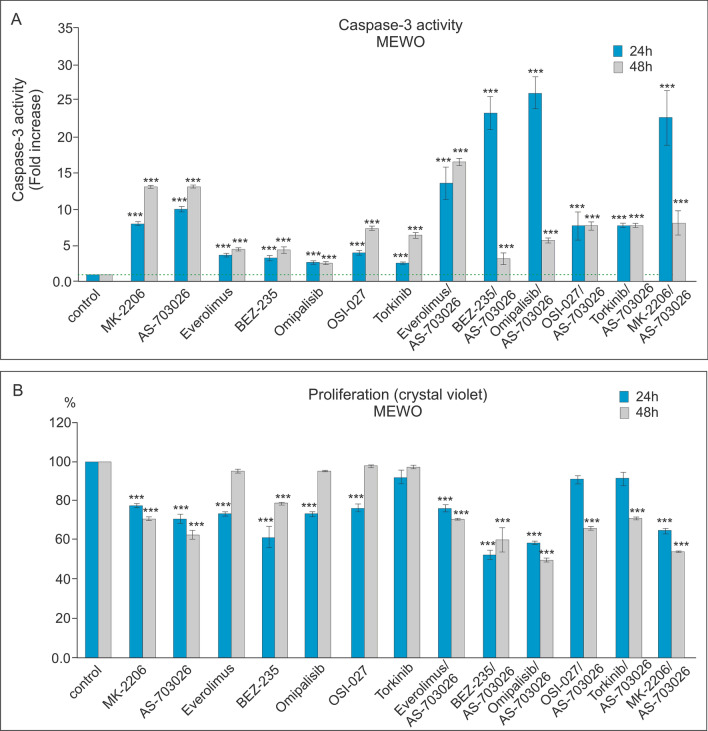
The effect of mTOR kinase inhibitors on caspase-3 activity (**A**) and cell proliferation in MEWO melanoma cell lines (**B**). Caspase-3 activity (**A**) and cell proliferation—crystal violet assay (**B**) were calculated from the mean values of three independent experiments. Each value was expressed as the ratio of caspase-3 activity or cell proliferation level to the control level; the control value was set at 1 for caspase-3 activity and 100% for cell proliferation. The data are presented as mean ± standard deviation; Statistical analyses were performed using one-way ANOVA with a post hoc Dunett test (Statistica 12.0 StatSoft); significant differences from control values are indicated as (*) *p* < 0.05, (**) *p* < 0.01, (***) *p* < 0.001

For the primary melanoma cell line—WM3211 (VGP), with a wild type for BRAF, PTEN, N-RAS, and CDK4, and with a mutation at position 576 in the c-KIT gene, caspase 3 activation compared to the control was several times lower than for the metastatic lines Mel-1359 and MEWO (Figs. 3, 4 and 5a).

In both metastasis-derived lines: Mel-1359 and MEWO, the use of single inhibitors and their combination with the MEK1/2-AS703026 inhibitor produced very similar trends of increased caspase 3 activity, with the increase slightly higher for the latter (Figs. 4, 5a).

The highest increases in caspase 3 activity were observed for the use of individual inhibitors compared to the control for the MEK1/2-AS-703026 inhibitor for the Mel-1359 line. The observed increase was approximately tenfold (*p* < 0.001) and 15-fold (*p* < 0.001) for 24 and 48 h (Fig. 4a), respectively. It was slightly lower for the AKT inhibitor—MK-2206: approximately 8 times (*p* < 0.001) for 24 h and 12 times (*p* < 0.001) for 48 h for the MEWO cell line (Fig. 5a). The remaining inhibitors showed increases in caspase activity of 3 to fivefold (*p* < 0.001) for 24 h and up to about sevenfold (*p* < 0.001) for 48 h (Figs. 4, 5a).

Treatment of melanoma cells with single inhibitors of PI3K and mTOR caused a decrease in proliferation compared to control cells, an effect that was the most pronounced for the Mel-1359 cell line (Figs. 3, 4 and 5b). Three inhibitors caused the highest decreases in proliferation during 24 h: BEZ-235, AS-703026, which saw a result of approximately 20% (*p* < 0.001) (Fig. 4b). The situation was similar for the 48 h period, during which time the decrease in proliferation reached 40% (*p* < 0.001) (Fig. 4b).

The use of the combination of PI3K and mTOR inhibitors with the MEK1/2 kinase inhibitor -AS-703026 resulted in the marked increase in caspase 3 activity compared to the use of single inhibitors (Figs. 3, 4 and 5a).

For both metastasis-derived lines: Mel-1359 and MEWO, a very similar trend of caspase 3 activation and a synergistic effect of the combination of inhibitors with the MEK1/2-AS703026 inhibitor (Figs. 4, 5a) were observed. For the primary line WM3211, an increase in caspase 3 activity was also observed after the use of the inhibitor combination, but these increases were up to 5 times lower than those of the metastatic lines and also showed a slightly different inhibitor preference (Figs. 3, 4 and 5a).

The largest and very similar activation of caspase 3 compared to the control occurred after the use of combinations of the MEK1/2-AS703026 inhibitor with dual PI3K kinase and mTOR kinase inhibitors: Omipalisib or BEZ-235.

The observed increase in caspase 3 activity was 30 and 27 times (*p* < 0.001) for the Mel-1359 line (Fig. 4a) for each of the combinations, respectively. A slightly lower increase in caspase 3 activity was observed (by approximately 20–25 times) for the use of the combination of inhibitors PI3K-MK-2206 and mTOR-everolimus together with the inhibitor MEK1/2—AS703026 (Fig. 4a).

On the other hand, the highest caspase 3 activation was achieved after cell treatment with the combination of mTOR inhibitors (everolimus or OSI-027 or Torkinib) with the MEK1/2 kinase inhibitor AS703026 (Fig. 4a). It was approximately 23 times (*p* < 0.001) after 48 h compared to the control. For the primary line WM3211, the highest, although small compared to other cell lines, were increases in caspase 3 activity after the combination of the MEK1/2 inhibitor-AS703026 with the following inhibitors: PI3K—MK2206 approximately 9 times (*p* < 0.001); the double inhibitors PI3K and mTOR BEZ-235 approximately 7 times (*p* < 0.001); and the inhibitor Omipalisib approximately 6 times (*p* < 0.001) (Fig. 3a).

The use of the combination of PI3K inhibitors, mTOR, with the MEK1/2 inhibitor AS703026 resulted in a marked decrease in melanoma cell proliferation (Figs. 3, 4 and 5b). These decreases reached even 50% for the combination with the inhibitor BEZ-235, Omipalisib, or MK-2206 (Figs. 3, 4 and 5b).

### Effect of mTOR kinase inhibitors on DNA fragmentation ELISA assay: detection of apoptosis

**Fig. 6:**
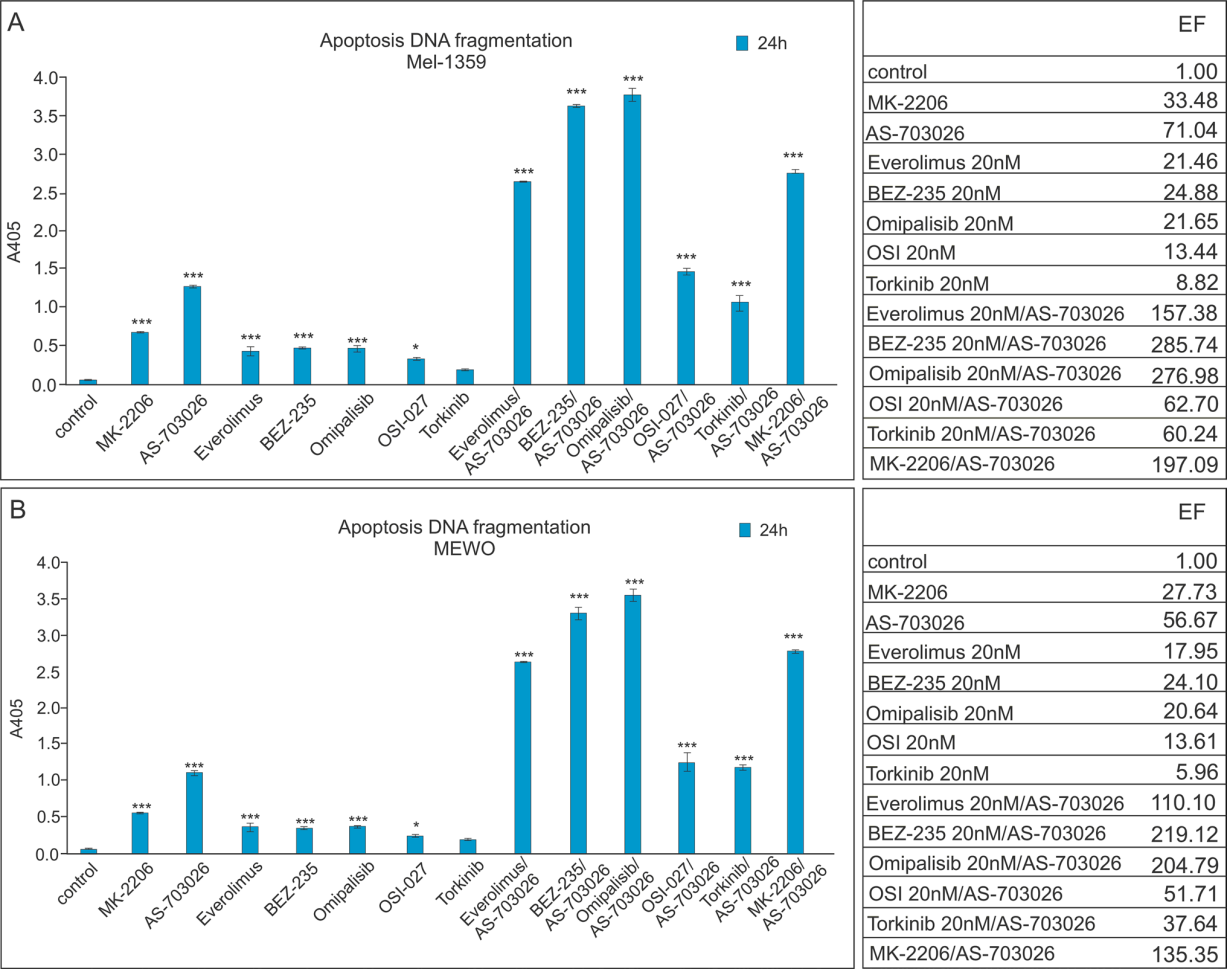
The effect of mTOR kinase inhibitors on melanoma cell apoptosis—Mel-1359 (**A**) and MEWO (**B**). The data are presented as mean ± standard deviation; Statistical analyses were performed using one-way ANOVA with a post hoc Dunett test (Statistica 12.0 StatSoft); significant differences from control values are indicated as (*) *p* < 0.05, (***) *p* < 0.001. EF, enrichment factor (calculated to estimate the fold increase in DNA fragmentation in treated samples with reference to the control)

For metastatic melanoma cell lines: Mel 1359 and MEWO, which showed the highest caspase 3 activity, the cell death detection ELISA assay that reflects DNA fragmentation in apoptotic cells was performed to verify the induction of apoptosis (Fig. 6).

As shown in Fig. 6, the activation profile of the apoptosis process for the Mel-1359 and MEWO melanoma cell lines, depending on the mTOR kinase inhibitor used, was quite similar and only slightly higher for the Mel-1359 cell line.

Except for the enrichment of the MEK1/2 inhibitor AS-703026 in the fragmented DNA content, none of the inhibitors used alone was considerably effective in triggering apoptosis.

The highest level of DNA apoptotic degradation was observed in response to concurrent application of the MEK 1/2 inhibitor—AS-703026 with dual inhibitors of PI3K kinase and mTOR kinase (Omipalisib or BEZ-235). A slightly lower effect was observed for the combination with the AKT inhibitor—MK-2206 or mTOR—everolimus (Fig. 6).

The apoptosis process was manifested by a significant increase in absorbance value compared to the untreated sample, with the enrichment factor EF (calculated to estimate the fold in-crease in DNA fragmentation in treated samples with reference to control one) for the Mel-1359 cell line reaching 276.98, and 285.74 for the MEK 1/2 inhibitor—AS-703026 combined with the dual PI3K kinase and mTOR kinase inhibitors Omipalisib or BEZ-235 (*p* < 0.001). A slightly lower effect was observed for the combination with the AKT inhibitor—MK-2206 or mTOR—everolimus, in which cases it reached 197.09 and 157.38, respectively (*p* < 0.001) (Fig. 6a). The result seems to suggest a synergistic effect of the applied agents.

Similarly, in the case of MEWO melanoma cells (Fig. 6b), each of the inhibitors used alone was hardly effective in induction of apoptosis, except the MEK1/2 inhibitor, AS-703026, manifested by a high EF value (~ 56). The use of the combination with the MEK1/2 AS-703036 inhibitor resulted in a slightly higher absorbance value, but the enrichment factor—EF values were slightly lower and were 204.79 and 219.12 (*p* < 0.001) for the combination with the dual inhibitors of PI3K kinase and mTOR kinase Omipalisib or BEZ-235, respectively. For the combination with mTOR—everolimus or the AKT inhibitor MK-2206, they reached 110.10 and 135.35 (*p* < 0.001), respectively (Fig. 6b).

**Fig. 7:**
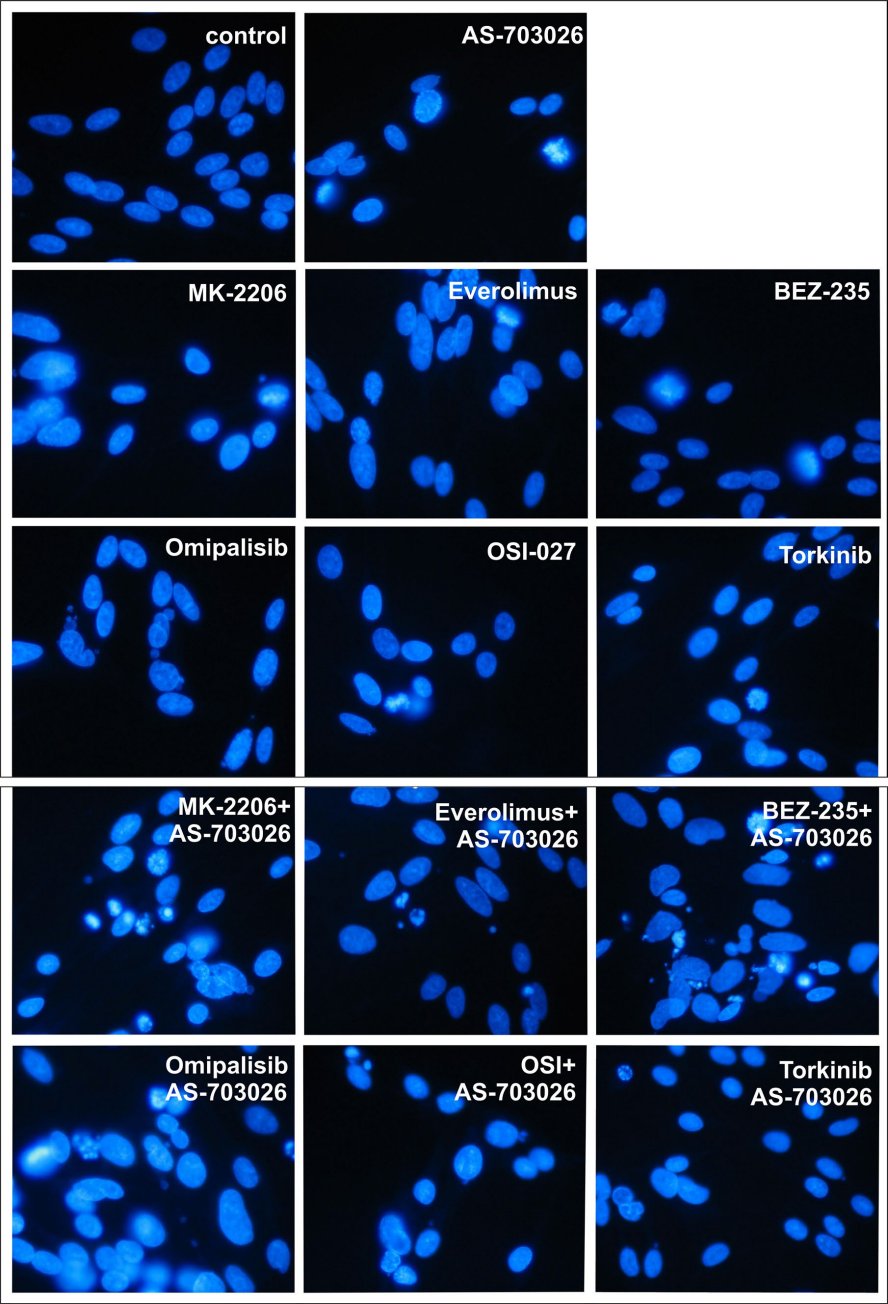
Morphological changes of MEWO cells after treatment with mTOR inhibitors for 24 h followed by DAPI staining. Apoptosis was confirmed by DAPI staining, which showed apparent changes in the nuclear morphology (chromatin condensation and nuclear fragmentation) of the MEWO cells. The concentrations of the inhibitors used are described in the Materials and Methods. The experiments were performed in triplicate

To confirm the morphological changes at the level of the cell nucleus and lysosomes, we stained the cells with DAPI (Fig. 7) and acridine orange (supplementary materials).

### Cytotoxicity assay

Cytotoxicity of selected kinase inhibitors: AKT—MK-2206 (2 μM), MEK1/2—AS-703026 (10 μM), mTOR—everolimus (20 nM), dual PI3K and mTOR inhibitor—BEZ-235 (20 nM), dual PI3K and mTOR inhibitor—Omipalisib (20 nM), mTOR1/2—OSI-027 (20 nM), mTOR—Torkinib (20 nM) was determined using Cytotoxicity Detection Kit LDH, Roche, Germany.

The inhibitors MK-2206, AS-703026, everolimus, BEZ-235, Omipalisib, OSI-027 and Torkinib showed no cytotoxicity effect at 24 h of treatment in any of the examined melanoma cell lines. LDH activity in culture medium in no case exceeded 3.9%; however, after 72 h of treatment, the effect of cytotoxicity was observed for the combination of inhibitors: BEZ-235 with AS-703026 (10.5%) and Omipalisib with AS-703026 (11%). We also observed an increase in lactate dehydrogenase activity for the Mel-1359 cell line for a combination of inhibitors: AS-703026 and MK-2206 (5.9%). No cytotoxic effect was observed for the primary melanoma cell line WM3211 (VGP), with a wild type for BRAF, PTEN, N-RAS, and CDK4, and mutation at position 576 in the c-KIT gene, even for long incubation times—72 h.

## Discussion

Serine threonine kinase, mTOR (mammalian target of rapamycin) is a protein that regulates many important intracellular processes. The research has shown that mTOR kinase is involved in transcription, translation, protein degradation, cytoskeleton organization, cell vesicle transport, and cell metabolism (Shimobayashi and Hall 2014; Mao and Zhang 2018; Conciatori et al. 2018a). Dysregulation of the mTOR kinase pathway is reported in the pathogenesis of numerous human diseases, including cancer, diabetes, cardiovascular and neurological diseases (Dormond 2019). mTOR kinase inhibitors, rapamycin and its rapalogs have been used in immunosuppressive therapy for solid organ transplantation for the last two decades, and there has been a growing interest in the use of these drugs in anticancer therapies (Janku et al. 2018; Conciatori et al. 2018b; Alammar et al. 2022).

In our previous studies on the treatment of melanoma cells with mTOR inhibitors, we have found that both rapamycin and everolimus had a significant impact on cell cycle regulation, cell proliferation (Ciołczyk-Wierzbicka et al. 2018), and invasive potential (Ciołczyk-Wierzbicka and Laidler 2018; Ciołczyk-Wierzbicka et al. 2020).

Promising results (Ciołczyk-Wierzbicka et al. 2019) regarding the nanomolar concentration of the mTOR inhibitor everolimus in combination with the inhibitor of MAP1/2 kinase (AS-703026) or AKT kinase (MK2206) in activation of the apoptosis process led to the continuation of research using various mTOR kinase inhibitors.

In the presented study, we investigated the effect of three generations of mTOR kinase inhibitors on the caspase 3 activation, apoptosis, and proliferation process, as well as inhibiting the expression of pro-survival proteins.

The research used: the first-generation mTOR kinase inhibitors such as everolimus or Torkinib, the second-generation inhibitors, ATP-competitive inhibitors of mTOR kinase that inhibit both mTORC1 and mTORC2—OSI-027, as well as some dual PI3K/mTOR inhibitors: BEZ-235 and Omipalisib.

### The first and the second generation of mTOR kinase inhibitors

Treatment of melanoma cells with a nanomolar concentration of mTOR kinase inhibitors such as everolimus or Torkinib showed statistically significant effects on caspase 3 activation and apoptosis, but this result was lower compared to other mTOR kinase inhibitors used. Studies carried out by many centers have confirmed that everolimus induces apoptosis in a variety of tumors cells: pancreatic cancer (Peng and Dou 2017), ovarian cancer (Guo et al. 2016), colon cancer (He et al. 2016), breast cancer (Du et al. 2018), N-RAS mutant neuroblastoma cell lines (Kiessling et al. 2016), and T-cell leukemia/lymphoma in long-term treatment (Darwiche et al. 2011). The effectiveness of the first-generation mTOR inhibitors is influenced by the fact that mTOR kinase is a complicated system that has various crosstalk’s with other signaling pathways. High concentrations of everolimus have also been found to induce a caspase-independent pathway—autophagy cell death (Nikoletopoulou et al. 2013; Lui et al. 2016; Paquette et al. 2018; Zeng et al. 2018).

The use of the OSI-027 inhibitor, which simultaneously blocks both the mTORC1 and mTORC2 subunits, resulted in a significant increase in caspase 3 activity, and in activation of the apoptosis process at a level similar to the first-generation mTOR inhibitors used. The second generation mTOR inhibitor OSI-027 is used in phase I gastric cancer, causing a decrease in proliferation and antiangiogenetic capacities, and antitumor activity in several cancers such as the prostate, renal, and bladder (Tian et al. 2019).

### The third generation of mTOR kinase inhibitors

The most pronounced effect among the mTOR kinase inhibitors used was obtained with dual PI3K/mTOR inhibitors: BEZ-235 and Omipalisib. The third-generation mTOR inhibitors such as BEZ-235 and Omipalisib have a bivalent structure to take advantage of the two docking sites allow resistance against the original compounds to be avoided, and therefore they are more effective compared to targeting mTOR alone (Tian et al. 2019; Hua et al. 2019). Dual PI3K and mTOR inhibitors, such as BEZ-235, have been used in studies on colorectal and breast cancer (Tian et al. 2019). According to Zhu et al. (2020), Omipalisib suppressed AKT activation, and downstream targets of mTOR significantly inhibited proliferation by inducing arrest of G0/G1 and activated the apoptosis process in esophageal squamous cell carcinoma (ESCC).

Another research group, Liu et al. (2015), also showed that Omipalisib suppressed the phosphorylation of the AKT, S6 and 4EBP1 proteins, thus inducing apoptosis and cell cycle arrest. The results demonstrated by Zhu et al. (2020) indicated that Omipalisib suppressed the activation of both PI3K/AKT/mTOR and ERK signaling in all ESCC cell lines, indicating that this inhibitor can suppress MAPK feedback activation. According to the research results obtained by Du et al. (2021), Omipalisib is an efficient sensitizer for DNA damage that causes induced cell death in vitro.

Among the single inhibitors used, the MEK1/2 inhibitor AS-703026 was the most effective in inducing apoptosis, measured by caspase-3 activation. Similar results were obtained for patients with multiple myeloma (Kim et al. 2010, 2017).

### Combinations mTOR inhibitors with MEK kinase inhibitors

Many research results indicate the efficacy of mTOR inhibitor monotherapy in some types of cancer; however, preclinical studies demonstrate strong rationales for combinatorial treatment with mTOR inhibitors and other drugs (Hua et al. 2019).

The signaling pathway associated with MAPK kinase has long been a promising target in the development of novel anticancer therapies. Inhibitors such as trametinib, cobimetinib, and binimetinib have been approved by the U.S. Food and Drug Administration for the treatment of BRAF V600E-mutated melanoma, non-small cell lung cancer, and anaplastic thyroid cancer (Lee et al. 2019). The highly selective, potent, noncompetitive allosteric ATP inhibitor of MEK1/2 kinase—AS-703026 (pimasertib) shows promise in inhibiting tumor growth, invasion, and activation of the caspase-3-dependent apoptosis process. Based on the information presented by the US National Library of Medicine (Clinical Trials.gov), clinical trials are underway to use this inhibitor in monotherapy and in combination with other drugs for locally advanced or metastasis cutaneous malignant melanoma, cancer, and hematopoietic and lymphoid cell neoplasms.

Unfortunately, in the case of many cancers, including breast cancer, inhibitors of the MAP kinase pathway show a short-term effect due to resistance that develops from multiple bypass feedback loops, including activation of the ATK or PI3K/AKT/mTOR pathways (Lee et al. 2019). Therefore, based on the reports from the literature and our own promising results, we decided to investigate the effect of three generations of mTOR inhibitors in the combination with an inhibitor of the MAP kinase pathway (AS-703026) on the apoptosis process in melanoma cells. For each of the mTOR kinase inhibitors used, the use of the combination with the MEK1/2 kinase AS-703026 inhibitor gave a clear synergistic effect both in caspase 3 activity, activation of the apoptosis process and inhibition of melanoma cell proliferation. This tendency was particularly evident in the activation of the apoptotic process. The process of apoptosis was confirmed by the ELISA test—DNA fragmentation as well as microscopic examination—DAPI staining which showed visible changes in the morphology of the nucleus (chromatin condensation and nuclear fragmentation) and staining with acridine orange (supplementary materials) changes at the lysosomal level.

The third-generation inhibitors such as BEZ-235 and Omipalisib in combination with the kinase inhibitor MEK1/2- AS-703026 caused the highest increase (about 3.5–4 times higher than the control) in the activation of the apoptotic process in melanoma cells. The data presented by Li et al. (2022) demonstrate that BEZ235, PP242 (Torkinib), and rapamycin exhibited antiproliferative, pro-apoptotic, and anti-invasive effects against renal cancer cells.

As a major member of dual PI3K/mTOR inhibitors, NVP-BEZ235 has undergone Phase I/II trials for the treatment of some cancers: prostate, breast, pancreatic, renal, or malignant solid tumor. Currently, Omipalisib is in a clinical trial for solid tumors; based on research results (Basu et al. 2018), this inhibitor could be considered for application in monotherapy or in combination therapy along with MEK162 (MEK1/2 kinase inhibitor) in patients with neurocutaneous melanocytosis. Basu et al. (2018) also showed that a dose-dependent increase in Omipalisib combined with a fixed dose of MEK162 (MEK1/2 inhibitor) was more effective than treatment with increasing doses of MEK162 combined with a fixed dose of Omipalisib. The use of high micromolar concentrations of Omipalisib induced the autophagy process in neurocutaneous melanocytosis cells (Basu et al. 2018).

The research results presented by Posch et al. (2013) confirmed the effectiveness of MEK/ERK and PI3K/mTOR inhibitors in the treatment of NRAS mutant melanoma.

Other research teams of Sweetlove et al. (2015) also showed the effectiveness of this combination in inhibiting cell growth in BRAF mutant melanoma.

An obvious but slightly lower effect (almost threefold) was observed for combination with the first-generation mTOR inhibitor everolimus or the AKT inhibitor MK2206 in combination with the MEK1/2 kinase inhibitor AS-703026.

Our previous research results (Ciołczyk-Wierzbicka et al. 2019) confirmed that the nanomolar concentration of the mTOR everolimus inhibitor in combination with the MEK1/2 inhibitor AS-703026 was effective in activating caspase 3 activity and the apoptosis process in the WM115 and WM266-4 melanoma cell lines.

On the other hand, high concentrations of this inhibitor, along with an inhibitor of the MAP kinase pathway, had a lower ability to activate apoptosis, possibly stimulating the autophagy process.

Many authors verify that everolimus induces the apoptosis process in cancer cells such as the pancreatic (Peng and Dou 2017), ovarian (Guo et al. 2016), colon (He et al. 2016), and breast (Du et al. 2018). Some tumors, particularly at high concentrations of the inhibitor, have also been shown to induce a caspase-independent pathway, autophagy cell death (Nikoletopoulou et al. 2013; Lui et al. 2016).

## Conclusions

The results of the studies using three generations of mTOR kinase inhibitors seem to be promising for activation of caspase 3, apoptosis process, and inhibition of melanoma cell proliferation. This makes it possible to use the third generation of mTOR inhibitors in particular in an anticancer therapy.

## Supplementary Information

Below is the link to the electronic supplementary material.

Supplementary file1 (DOCX 2631 kb)
