# Correction to: Three generations of mTOR kinase inhibitors in the activation of the apoptosis process in melanoma cells

**DOI:** 10.1007/s12079-023-00764-9

**Published:** 2023-05-09

**Authors:** Dorota Ciołczyk-Wierzbicka, Agnieszka Krawczyk, Marta Zarzycka, Grzegorz Zemanek, Karol Wierzbicki

**Affiliations:** 1 Chair of Medical Biochemistry, Jagiellonian University Medical College, Ul. Kopernika 7, Kraków, 31-034 Poland; 2 Department of Cardiovascular Surgery and Transplantology, Institute of Cardiology, John Paul II Hospital, Jagiellonian University, Ul. Prądnicka 80, 31-202, Kraków, Poland

**Correction to: Journal of Cell Communication and Signaling**

10.1007/s12079-023-00748-9

In this article the author name Dorota Ciołczyk-Wierzbicka was incorrectly written as Dorota Ciołczyk-Wierzbica.

The original article has been corrected.

