# Supplementary material for: Three generations of mTOR kinase inhibitors in the activation of the apoptosis process in melanoma cells
Source: J Cell Commun Signal. 2023 Apr 25;17(3):975–89. doi: 10.1007/s12079-023-00748-9 (PMC10409930; doi:10.1007/s12079-023-00748-9)
Supplement: Supplementary file 1 — Supplementary file1 (DOCX 2631 kb) [file 12079_2023_748_MOESM1_ESM.docx]

**Three generations of mTOR kinase inhibitors in the activation of the apoptosis process in melanoma cells.**

Dorota Ciołczyk-Wierzbicka^1^, Agnieszka Krawczyk^1^, Marta Zarzycka^1^ , Grzegorz Zemanek^1^ and Karol Wierzbicki^2^

^1^ Chair of Medical Biochemistry, Jagiellonian University Medical College, Kraków, Poland;

ul. Kopernika 7, 31-034 Kraków Tel: +4812 4227400; Fax: +4812 4223272;

E-mail: dorota.ciolczyk-wierzbicka@uj.edu.pl

^2^ Department of Cardiovascular Surgery and Transplantology, Institute of Cardiology, Jagiellonian University, John Paul II Hospital; ul. Prądnicka 80, 31-202 Kraków, Poland

***** Correspondence: Dorota Ciołczyk-Wierzbicka, Chair of Medical Biochemistry, Jagiellonian University Medical College, Kraków, Poland; ul. Kopernika 7, 31-034 Kraków, Poland

Tel: +4812 422 74 00; Fax: +4812 422 32 72;

E-mail: [dorota.ciolczyk-wierzbicka@uj.edu.pl](mailto:dorota.ciolczyk-wierzbicka@uj.edu.pl)

**Keywords:** mTOR inhibitors; apoptosis; caspase-3 activity; proliferation; melanoma; immunosuppressive treatment


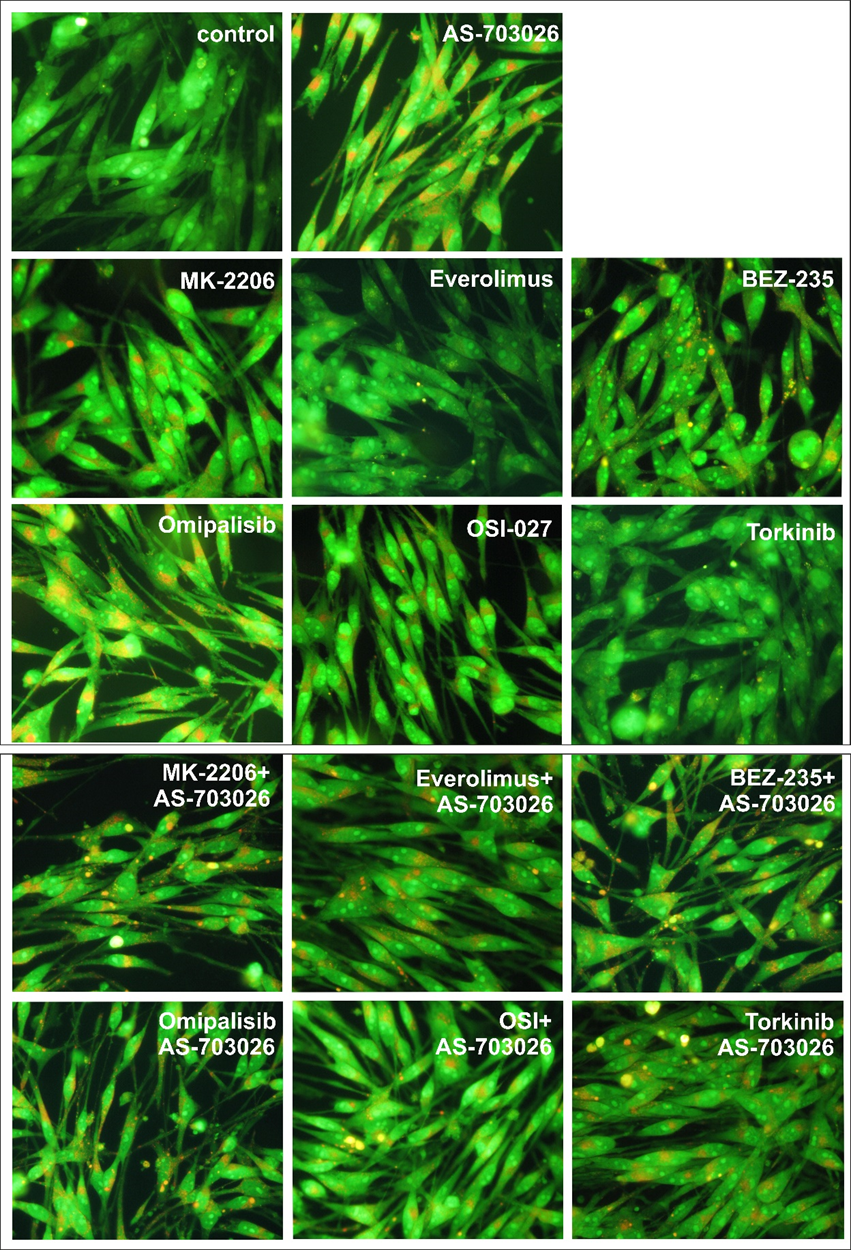


Figure S1. Morphological changes of MEWO cells after treatment with mTOR inhibitors for 24 h followed by acridine orange staining. The concentrations of the inhibitors used are described in the Materials and Methods. The experiments were performed in triplicate.

**Materials and Methods**

**Acridine orange staining assay**

Acridine Orange hydrochloride (Sigma) staining was carried out according to the method described by Yoshida et al. 2013.

Kiyoshima T, Yoshida H, Wada H, Nagata K, Fujiwara H, Kihara M, Hasegawa K, Someya H, Sakai H. Chemoresistance to concanamycin A1 in human oral squamous cell carcinoma is attenuated by an HDAC inhibitor partly via suppression of Bcl-2 expression. PLoS One. 2013 Nov 20;8(11):e80998. doi: 10.1371/journal.pone.0080998. PMID: 24278362; PMCID: PMC3835574.
